# What Do We Feed to Food-Production Animals? A Review of Animal Feed Ingredients and Their Potential Impacts on Human Health

**DOI:** 10.1289/ehp.9760

**Published:** 2007-02-08

**Authors:** Amy R. Sapkota, Lisa Y. Lefferts, Shawn McKenzie, Polly Walker

**Affiliations:** 1 Johns Hopkins Center for a Livable Future, Bloomberg School of Public Health, Baltimore, Maryland, USA; 2 Maryland Institute for Applied Environmental Health, College of Health and Human Performance, University of Maryland, College Park, Maryland, USA; 3 Lisa Y. Lefferts Consulting, Nellysford, Virginia, USA

**Keywords:** animal feed, animal waste, concentrated animal feeding operations, fats, human health effects, nontherapeutic antibiotics, rendered animals, roxarsone, zoonoses

## Abstract

**Objective:**

Animal feeding practices in the United States have changed considerably over the past century. As large-scale, concentrated production methods have become the predominant model for animal husbandry, animal feeds have been modified to include ingredients ranging from rendered animals and animal waste to antibiotics and organoarsenicals. In this article we review current U.S. animal feeding practices and etiologic agents that have been detected in animal feed. Evidence that current feeding practices may lead to adverse human health impacts is also evaluated.

**Data sources:**

We reviewed published veterinary and human-health literature regarding animal feeding practices, etiologic agents present in feed, and human health effects along with proceedings from animal feed workshops.

**Data extraction:**

Data were extracted from peer-reviewed articles and books identified using PubMed, Agricola, U.S. Department of Agriculture, Food and Drug Administration, and Centers for Disease Control and Prevention databases.

**Data synthesis:**

Findings emphasize that current animal feeding practices can result in the presence of bacteria, antibiotic-resistant bacteria, prions, arsenicals, and dioxins in feed and animal-based food products. Despite a range of potential human health impacts that could ensue, there are significant data gaps that prevent comprehensive assessments of human health risks associated with animal feed. Limited data are collected at the federal or state level concerning the amounts of specific ingredients used in animal feed, and there are insufficient surveillance systems to monitor etiologic agents “from farm to fork.”

**Conclusions:**

Increased funding for integrated veterinary and human health surveillance systems and increased collaboration among feed professionals, animal producers, and veterinary and public health officials is necessary to effectively address these issues.

Animal-based food products derived from cattle, swine, sheep, poultry, and farmed fish constitute a significant portion of the current U.S. diet. In 2003, the U.S. per capita consumption of total meats (including beef, pork, veal, lamb, poultry, fish, and shellfish) was 90.5 kg/year [U.S. Department of Agriculture (USDA) 2005a]. Data from animal-production researchers demonstrate that the quality of these products is directly related to animal feeding practices (Capucille et al. 2004; Gatlin et al. 2003; Zaghini et al. 2005). Therefore, given the high consumption of animal-based food products in the United States, the ingredients used in animal feed are fundamentally important in terms of both the quality of the resulting food products and the potential human health impacts associated with the animal-based food-production chain.

In the early 1900s, animals produced for food in the United States were raised on small family farms where cows predominantly grazed on pasture and young chickens were fed primarily a corn-based diet (Erf 1907). However, in the past 60 years, farms and animal feed formulations have undergone significant changes. Small family-owned and -operated farms have been replaced almost entirely by a system of large-scale operations where individual farmers contract with vertically integrated corporations. High rates of food production have been achieved through these systems in which the scale of operations requires the high throughput generation of animals for processing. Animals are raised in confinement and fed defined feeds that are formulated to increase growth rates and feed-conversion efficiencies. These present day animal feeds contain mixtures of plant-based products, as well as other ingredients ranging from rendered animals and animal waste to antibiotics and organoarsenicals. The inclusion of these ingredients in animal feeds can result in the presence of a range of biological, chemical, and other etiologic agents in feed that can affect the quality and safety of animal-based food products and pose potential risks to human health.

Since December 2003, when the first U.S. case of bovine spongiform encephalopathy (BSE) was identified in a dairy cow in Washington State, there has been increased attention from veterinary and public health professionals regarding the quality and safety of U.S. animal feed, as well as the safety of subsequent animal-based food products. Yet, the focus of such attention is often limited to one particular facet of animal feed and its associated animal or human health effect (i.e., the impact of rendered animals in feed formulations on the risk of BSE, the impact of bacterial contamination of animal feed on human bacterial illnesses). However, to begin to understand the broad range of potential human health impacts associated with current animal feeding practices, it is necessary to examine the full spectrum of feeding practices and assess their potential human health implications collectively.

In this article we review U.S. animal feed–production practices; animal feed ingredients; and biological, chemical, and other etiologic agents that have been detected in animal feed. In addition, we evaluate evidence that current feeding practices may be associated with adverse human health impacts, and address the data gaps that prevent comprehensive assessments of human health risks associated with animal feed.

## U.S. Animal Feed Production

The U.S. animal feed industry is the largest producer of animal feed in the world (Gill 2004). In 2004, over 120 million tons of primary animal feed, including mixes of feed grains, mill by-products, animal proteins, and microingredient formulations (i.e., vitamins, minerals, and antibiotics) were produced in the United States (Gill 2004). In the same year, the United States exported nearly $4 billion worth of animal feed ingredients (International Trade Centre 2004).

The structure of the U.S. animal feed industry is complex, with a multitude of industries and individual producers contributing to the production, mixing, and distribution of feed ingredients and complete feed products. However, there are a few firms that play principal roles in the manufacture of U.S. feeds, including feed mills, rendering plants, and protein blenders [General Accounting Office (GAO) 2000]. Feed mills combine plant- and animal-based feed ingredients to produce mixes designed for specific animal species (GAO 2000). Rendering plants transform slaughter by-products and animals that are unsuitable for human consumption into animal feed products using grinding, cooking, and pressing processes (GAO 2000; National Renderers Association Inc. 2005a). Protein blenders mix processed plant- and animal-based protein ingredients from many sources into animal feeds (GAO 2000). Once animal feed ingredients are mixed, an estimated 17,500 U.S. animal feed dealers distribute the final feed products to individual feeding operations (Feedstuffs 2005).

## Animal Feed Ingredients and Feeding Practices

Animal feed ingredients that constitute complete feed products are derived from a multitude of raw materials of plant and animal origin, as well as pharmaceutical and industrial sources. Specific feed ingredients vary depending upon the animal (i.e., poultry, swine, cattle); Table 1 provides an overview of feed ingredients that are legally permitted and used in U.S. animal feed. More specific information about feed ingredients listed in Table 1 is available in the *Official Publication* of the Association of American Feed Control Officials, Inc. (AAFCO), which is published annually (AAFCO 2004), and in Lefferts et al. (2006). In the present review we focus on feed ingredients listed in Table 1 that raise specific concerns for public health, including rendered animal products, animal waste, plant- and animal-based fats, antibiotics, and metals.

### Rendered animal products

In 2003, the U.S. rendering industry produced > 8 million metric tons of rendered animal products, including meat and bone meal, poultry byproduct meal, blood meal, and feather meal (National Renderers Association Inc. 2005b). Most of these products were incorporated into animal feed. However, data concerning the specific amounts of rendered animal protein that are used in animal feed are difficult to obtain because the information is neither routinely collected at the federal or state level nor reported by the rendering industry. The latest available data, collected by the USDA in 1984, estimated that > 4 million metric tons of rendered animal products were used as animal feed ingredients (USDA 1988). Oftentimes these ingredients are listed on animal feed labels as “animal protein products.” Thus, it is difficult to discern precisely which animal protein products are included in a particular animal feed product (Lefferts et al. 2006).

### Animal waste

Another major animal protein–based feed ingredient is animal waste, including dried ruminant waste, dried poultry litter, and dried swine waste (AAFCO 2004; Haapapuro et al. 1997). As with rendered animal products, there are no national data on the total amounts of animal waste included in animal feeds, although some states have collected limited data concerning this practice. In 2003, it was estimated that approximately 1 million tons of poultry litter were produced annually in Florida, and an estimated 350,000 tons of this litter were available for use in feed (Dubberly 2003). Yet, information concerning the precise amount of this “available” poultry litter that was actually incorporated into Florida animal feed was unavailable.

Recycling animal waste into animal feed has been practiced for > 40 years as a means of cutting feed costs. However, the U.S. Food and Drug Administration (FDA) does not officially endorse the use of animal waste in feed and has issued statements voicing the agency’s concern about the presence of pathogens and drug residues in animal waste, particularly poultry litter (FDA 1998). In line with these concerns, the AAFCO, an organization that develops guidelines for the safe use of animal feeds, advises that processed animal waste should not contain pathogenic microorganisms, pesticide residues, or drug residues that could harm animals or eventually be detected in animal-based food products intended for human consumption (AAFCO 2004). Nonetheless, these guidelines are not adequately enforced at the federal or state level.

### Plant- and animal-based fats

In addition to animal protein–based ingredients, fats originating from both plant and animal sources are included in animal feed (Table 1) and may contain contaminants such as dioxins and polychlorinated biphenyls (PCBs), which are harmful to human health. In 1988, the USDA (1988) reported that approximately 1.3 million metric tons of fats were used in the production of U.S. primary animal feed. Unfortunately, as with many other animal feed ingredients, we were not able to obtain recent data. Yet, because as much as 8% of feed could be composed of fats alone (Schmidt 2004), the quality (i.e., contaminant levels) of both plant and animal fats used in animal feed could be important factors in the ultimate safety of animal-based food products.

### Antibiotics

The use of antibiotics in animal feed is also a public health concern. Antibiotics are administered at nontherapeutic levels in feed and water to promote growth and improve feed efficiency. This practice has been shown to select for antibiotic resistance in both commensal and pathogenic bacteria in *a*) the animals themselves (Aarestrup et al. 2000; Bager et al. 1997; Gorbach 2001; Wegener 2003); *b*) subsequent animal-based food products (Hayes et al. 2003; White et al. 2001); and *c*) water, air, and soil samples collected around large-scale animal feeding operations (Chapin et al. 2005; Chee-Sanford et al. 2001; Gibbs et al. 2006; Jensen et al. 2002).

Although the use of nontherapeutic levels of antibiotics in animal feed is approved and regulated by the FDA (2004), there is no U.S. data collection system regarding the specific types and amounts of antibiotics that are used for this purpose. In response to this significant data gap, several estimates of nontherapeutic antibiotic usage have been published based on USDA livestock production data and FDA antibiotic usage regulations. For example, Mellon et al. (2001) estimated that as much as 60–80% of antibiotics produced in the United States are administered in feed to healthy livestock at nontherapeutic levels. Many of these antibiotics are the same compounds that are administered to humans in clinical settings, and include tetracyclines, macrolides, streptogramins, and fluoroquinolones (FDA 2004). Additional information regarding the types and amounts of antibiotics used in U.S. livestock is available in AAFCO (2004), FDA (2004), and Mellon et al. (2001).

### Metals

Metal compounds are also administered in animal feeds, and the compounds currently added to both swine and poultry feeds that are particularly concerning from a public health perspective are organoarsenicals. The most commonly used organoarsenical, roxarsone (4-hydroxy-3-nitrobenzenearsenic-acid), is administered to feeds at concentrations ranging from 22.7 g/ton to 45.4 g/ton to promote growth and improve feed efficiency (Chapman and Johnson 2002). When used in combination with ionophores, roxarsone also act as a co-coccidiostat to control intestinal parasites (Chapman and Johnson 2002). Once roxarsone is ingested by animals, the parent compound can be degraded into inorganic arsenite (As^III^) and inorganic arsenate (As^V^) in animal digestive tracts and animal waste (Arai et al. 2003; Stolz et al. 2007). Both As^III^ and As^V^ are classified by the U.S. Environmental Protection Agency (U.S. EPA) as group A human carcinogens (U.S. EPA 1998). Many other metallic compounds are also mixed into feeds, including copper, manganese, magnesium, and zinc compounds, as well as metal amino acid complexes (AAFCO 2004).

## Biological, Chemical, and Other Etiologic Agents Detected in Animal Feed

Because of current animal feeding practices, biological, chemical, and other etiologic agents have been detected in animal feeds (Table 2) (Hinton 2000; Orriss 1997). These agents include bacterial pathogens, antibiotic-resistant bacteria, prions, metals, mycotoxins, polychlorinated dibenzo-*p*-dioxins (PCDDs), polychlorinated dibenzofurans (PCDFs), and PCBs (Crump et al. 2002; Dargatz et al. 2005; Eljarrat et al. 2002; Lasky et al. 2004; Moreno-Lopez 2002).

### Bacteria

There is substantial evidence that U.S. animal feeds are often contaminated with important human foodborne bacterial pathogens such as *Salmonella* spp. (Crump et al. 2002; Davis et al. 2003; Krytenburg et al. 1998) and *Escherichia coli,* including *E. coli* O157:H7 (Dargatz et al. 2005; Davis et al. 2003; Lynn et al. 1998; Sargeant et al. 2004). Studies of *Salmonella* spp. indicate that this pathogen can enter animal feeds at several points throughout the feed production process, including the primary production of feed ingredients, milling, mixing, and/or storage (Maciorowski et al. 2006). However, a main source of *Salmonella* spp. contamination in animal feed is often the specific feed ingredients (originating from both plant and animal sources) that are combined at feed mills (Coma 2003; Davis et al. 2003). Once complete feeds are delivered to animal feeding operations, additional contamination with *Salmonella* spp. can occur if the feeds are disturbed by insects and wild birds or animals that harbor *Salmonella* spp. (Maciorowski et al. 2006).

One of the first reports of the presence of non-typhi serotypes of *Salmonella enterica* in U.S. poultry feed samples was published by Edwards et al. (1948). Since then, researchers have detected *S. enterica* in a variety of feed ingredients and complete feed products; however, the results from these studies have been variable. McChesney et al. (1995) found that 56% of 101 animal protein–based feed samples collected from 78 rendering plants and 36% of 50 vegetable protein–based feed samples collected from 46 feed mills were positive for *S. enterica*. In contrast, Krytenburg et al. (1998) detected *S. enterica* in 9.8% of 295 feed samples from commercially prepared cattle feeds present at feedlots in the northwestern United States. More recently, Dargatz et al. (2005) detected *Salmonella* spp. in 24% of 175 samples of mixed feed collected from a cattle feedlot in Colorado, and another study identified *Salmonella* spp. in 14% of meat and bone meal samples collected from a poultry feed mill (Hofacre et al. 2001).

*E. coli* also has been detected in animal feeds (Dargatz et al. 2005; Davis et al. 2003; Lynn et al. 1998; Sargeant et al. 2004). In a study by Lynn et al. (1998), 30.1% of 209 samples of cattle feed—collected from 13 dairies, 1 calf research facility, and 4 feed mills—were positive for *E. coli*; none of the samples were positive for *E. coli* O157:H7. In contrast, Sargeant et al. (2004) isolated *E. coli* O157:H7 from 14.9% of 504 cattle feed samples collected in the midwestern United States. More recently, Dargatz et al. (2005) recovered *E. coli* from 48.2% of 1,070 cattle feed samples collected in Colorado.

### Antibiotic-resistant bacteria

A limited number of studies also have detected antibiotic-resistant bacteria in animal feeds. Schwalbe et al. (1999) tested poultry feeds and isolated *Enterococcus faecium* that were resistant to vancomycin, gentamicin, streptomycin, and ampicillin. In a study of cattle feed ingredients, Dargatz et al. (2005) found that 38.7% of 514 *E. coli* isolates were resistant to cephalothin, 24.7% were resistant to ampicillin, 16.6% were resistant to cefoxitin, and 12.1% were resistant to amoxicillin/clavulanic acid. Among the 57 *Salmonella* spp. recovered from cattle feed ingredients, 34.5% were resistant to sulfamethoxazole, 15.5% were resistant to cephalothin, 13.8% were resistant to cefoxitin, 12.1% were resistant to ampicillin, 10.3% were resistant to amoxicillin/clavulanic acid, and 10.3% were resistant to ceftiofur (Dargatz et al. 2005). In the same study, the authors also detected multiple antibiotic-resistant *E. coli* and *Salmonella* spp.

In another study, 165 rendered animal protein products originating from poultry, cattle, and fish were sampled from a poultry feed mill and tested for antibiotic-resistant bacteria (Hofacre et al. 2001). Eighty-five percent of all feed ingredients sampled contained bacteria resistant to one or more of the following four antibiotics: ampicillin, amoxicillin, clavulanic acid, and cephalothin. Poultry meal and bone and meat meal (nonpoultry) samples represented the greatest number of feed ingredient samples containing bacteria resistant to five or more antibiotics (Hofacre et al. 2001).

### Prions

In addition to bacteria, animal feeds (in particular, cattle feeds) can be contaminated with the infectious agent associated with BSE (Gizzi et al. 2003). BSE, which is commonly referred to as mad cow disease, belongs to a group of progressively degenerative neurologic diseases called transmissible spongiform encephalopathies (TSEs) (Deslys and Grassi 2005; Smith 2003). The causative agent of TSEs is believed to be an infectious proteinaceous entity called a prion, which is composed largely of a protease-resistant misfolded protein (PrP^Sc^). Infectious prions can be present in animal feed as a result of using rendered animal products from diseased animals as feed ingredients. Although prions may be present in all body tissues of diseased animals, it is generally acknowledged that prions accumulate in highest concentrations in central nervous system tissues (GAO 2002; Smith 2003) that are referred to as specified risk materials (SRMs). As defined by the USDA Food Safety Inspection Service (USDA 2005b), SRMs include the skull, brain, eyes, parts of the vertebral column, spinal cord, trigeminal ganglia, and dorsal root ganglia of cattle > 30 months of age, as well as the tonsils and distal ileum of all cattle. In 1997, the FDA banned SRMs from use in cattle and other ruminant feed (GAO 2002). Nonetheless, SRMs were allowed to be incorporated into feeds for nonruminants (including poultry), and subsequent waste products from non-ruminants are still permitted in ruminant feeds (USDA 2005b).

As of yet, there are no definitive tests for BSE infectivity in live animals (before symptoms appear) (Deslys and Grassi 2005; GAO 2002). However, a number of rapid screening tests based on ELISA or Western blot analyses have been approved for post-mortem BSE testing in cattle. Currently, the USDA is conducting a national BSE testing program; yet, only high-risk cattle are included in the program and there are no plans to test animal feed samples (that could include animal protein from asymptomatic rendered animals) in this surveillance effort (USDA 2004). A variety of tests do exist for the detection of animal tissues (in general) in animal feed, including microscopic analyses, polymerase chain reaction, immunoassay analyses, and near infrared spectroscopy (Gizzi et al. 2003); nonetheless, these methods are not robust enough to distinguish between bovine products that are permitted in ruminant feeds (i.e., milk and blood) and bovine products that are prohibited from ruminant feeds (GAO 2002; Momcilovic and Rasooly 2000).

### Mycotoxins

Mycotoxins unintentionally appear in animal feed as a result of the inadvertent use of mycotoxin-contaminated feed ingredients such as cereal grains. Mycotoxins are toxic secondary metabolites produced by filamentous fungi (molds) that can invade crops while they are growing in the field and while they are being processed and stored (Bhat and Vasanthi 1999). The mycotoxins of greatest agricultural and public health significance include aflatoxins, ochratoxins, trichothecenes, fumonisins, zearalenone, and ergot alkaloids (Cleveland et al. 2003; Hussein and Brasel 2001). The International Agency for Research on Cancer (IARC 1993) has classified aflatoxin as a group 1 human carcinogen; ochratoxins and fumonosins as group 2B possible human carcinogens; and trichothecenes and zearalenone as noncarcinogens (group 3). Trichothecenes are highly toxic to humans, and zearalenones are recognized endocrine disruptors (IARC 1993).

Because of these classifications, the FDA (2001) has established recommended maximum levels for aflatoxins and fumonisins in animal feed. For swine, ruminants, and poultry, the recommended maximum levels of total fumonisins in complete feeds are 10, 30, and 50 μg/g, respectively (FDA 2001). Nonetheless, although recommended maximum levels exist, it is very difficult to determine the extent of mycotoxin contamination in feedstuffs. Mycotoxins are unevenly distributed in feed, introducing a significant amount of sampling error into sample analyses (Hussein and Brasel 2001). In addition, there is wide geographic and temporal variability in the occurrence of mycotoxins in animal feed that is partially attributed to environmental factors (i.e., rainfall, humidity) (Hussein and Brasel 2001).

### PCDDs, PCDFs, and PCBs

Other unintentional contaminants of animal feed include dioxins, such as PCDDs and PCDFs, and PCBs (Eljarrat et al. 2002). Based on human and animal studies, IARC (1997) has classified 2,3,7,8-tetrachlorodibenzo-*p*-dioxin (TCDD, the most potent dioxin congener) as a group 1 human carcinogen. The presence of PCDDs, PCDFs, and PCBs in the environment is largely attributed to human activities, including the incineration of plastics and industrial processes involving chlorinated compounds. When dioxins and PCBs are released into the environment, they can contaminate plant-based animal feeds through a variety of pathways, including the airborne deposition of particles onto plant and soil surfaces (Fries 1995). When these lipophilic compounds are ingested by food-production animals, they bioaccumulate in fat tissues, making the use of rendered animal fats and oils in animal feed a significant source of exposure to dioxins and PCBs among food-production animals (Eljarrat et al. 2002; Institute of Medicine 2003).

The most severe example of dioxin- and PCB-contaminated animal feed occurred in Belgium in 1999 (van Larebeke et al. 2001). A fat-melting company inadvertently incorporated mineral oil containing 40–50 kg of PCBs and approximately 1 g of dioxins into a mixture of animal-based fats that were subsequently distributed to 10 animal feed producers, resulting in approximately 500 tons of contaminated feed (van Larebeke et al. 2001). The levels (mean ± SD) of PCBs and dioxins detected in contaminated animal feed were 1658.4 ± 23584.4 ng/g of fat and 2319.8 ± 3851.9 pg international toxic equivalents (I-TEQs)/g of fat, respectively, and resulted in elevated levels of these compounds in animal-based food products such as eggs, poultry, and pork (van Larebeke et al. 2001). Beyond this incident, several European studies have described elevated levels of PCDDs and PCDFs in eggs from free-range chickens raised on dioxin-contaminated soils (Schoeters and Hoogenboom 2006). In the United States, a significant episode of dioxin-contaminated feed occurred in 1997 (Hayward et al. 1999). Elevated levels of dioxin were detected in chicken eggs and farm-raised catfish; the source of contamination was traced to ball clay that was used as an anticaking agent and pelleting aid in poultry feed, bovine pellets, and catfish nuggets (Hayward et al. 1999). Once the source of contamination was identified, the FDA issued a statement to producers requesting the elimination of ball clay from feed ingredients (FDA 1997).

Aside from these accidents, little data have been generated in the United States concerning levels of dioxins and PCBs that are typically found in U.S. livestock feed. However, numerous studies have documented higher levels of PCBs and dioxins in farmed salmon versus wild-caught salmon, and these elevated contaminant levels have been attributed to contaminated commercial salmon feed (Easton et al. 2002; Hites et al. 2004). For example, Easton et al. (2002) detected total PCBs at mean concentrations of 51,216 pg/g and 5,302 pg/g in farmed and wild-caught salmon, respectively, and a mean concentration of 65,535 pg/g in commercial salmon feed.

## Potential Human Health Impacts Associated with Etiologic Agents Present in Animal Feed

To determine whether the presence of biological, chemical, and other etiologic agents in animal feed affects human health, it is necessary to integrate data from robust veterinary and human health surveillance systems that monitor agents in feed, health effects in animals, contaminants in animal-based food products, and illnesses in humans. However, to date, these integrated systems are largely lacking in the United States. Thus, the current evidence regarding human health risks associated with U.S. animal feed has been obtained mostly from isolated case reports and outbreaks published in the peer-reviewed literature. Some of this evidence is described below and outlined in Table 2; yet, as a result of significant data gaps, this information may represent only a small proportion of potential human health risks associated with animal feed.

### Bacterial infections

Crump et al. (2002) cited the emergence of *S. enterica* serotype Agona infections in humans in the United States as an example of human foodborne bacterial infections that have been definitively traced to contaminated animal feed. *S. enterica* serotype Agona infections are characterized by fever, diarrhea, abdominal cramps, and vomiting; the illness can be fatal in infants, the elderly, and immunocompromised individuals. Before 1970, only two cases of *S. enterica* serotype Agona infection had been reported in the United States (Crump et al. 2002). However, by 1972, *S. enterica* serotype Agona was among the top 10 most frequently isolated *S. enterica* serotypes from human infections (Crump et al. 2002). An epidemiologic study identified the source of these *S. enterica* serotype Agona infections as chicken meat that originated from a poultry facility where Peruvian fish meal was used as a feed ingredient (Clark et al. 1973; Crump et al. 2002). Clark et al. (1973) found that the fish meal had been contaminated with *S. enterica* serotype Agona before being incorporated into the poultry feed. Crump et al. (2002) estimated that since the introduction of *S. enterica* serotype Agona in poultry feed in 1968, this serotype has likely caused over 1 million human bacterial illnesses in the United States.

Besides the *S. enterica* serotype Agona example, there are insufficient data available to understand the extent to which other human bacterial illnesses arising in the United States are the result of contaminated animal feed. Outbreaks of human bacterial illness often can be traced to food-production animals or facilities; however, because of surveillance inadequacies, it is difficult to determine the initial source of bacterial contamination (i.e., animal feed or other factors) within the animal-production environment. Moreover, unlike *S. enterica* serotype Agona—which could be traced to poultry feed in 1968 because it was a newly identified serotype—it is much more difficult to understand the associations between more common, widespread serotypes or bacterial species present in animal feed and human illnesses. Nevertheless, with the use of annual U.S. foodborne illness data, estimates concerning the contributions of contaminated animal feed to human bacterial illnesses have been made (Angulo 2004). Based on the assumptions that food-production animals are the source of 95% of human nontyphoidal *Salmonella* cases and that 10% of food-production animals are infected by *Salmonella* spp. through the ingestion of contaminated animal feed, it has been estimated that approximately 134,000 cases of human nontyphoidal salmonellosis (including 55 deaths and 1,560 hospitalizations) could be attributed to contaminated animal feed each year (Angulo 2004).

### Antibiotic-resistant bacterial infections

Similar to the challenge of determining whether human bacterial illnesses are associated with contaminated animal feed, there are insufficient data available to determine the percentage of antibiotic-resistant human bacterial infections that are attributed to animal feeding practices versus practices and behaviors occurring in human clinical settings. This is largely the result of *a*) insufficient data on agricultural antibiotic usage in the United States; *b*) insufficient surveillance data concerning the dissemination of antibiotic-resistant isolates from food-production animals to humans; *c*) insufficient investigations regarding the original sources of resistant infections diagnosed in hospital settings; and *d*) underreporting of community-acquired antibiotic-resistant bacterial infections.

Nonetheless, there is evidence that antibiotic-resistant bacteria can be transmitted from swine and poultry to humans (Aarestrup et al. 2000). Sorensen et al. (2001) reported that after the ingestion of antibiotic-resistant *Enterococcus faecium* originating from contaminated chicken and pork, the resistant bacterium can be isolated from the stool of infected individuals for up to 2 weeks, indicating that antibiotic-resistant *E. faecium* can survive and multiply in the human gastrointestinal tract. In addition, there is strong temporal evidence suggesting that some domestically acquired antibiotic-resistant bacterial infections in humans emerged in the United States only after the approval of specific human antibiotics for use in animal feed or water. For example, prior to 1985 there were little or no fluoroquinolone-resistant *Campylobacter jejuni* isolated from either poultry or humans in the United States (Smith et al. 1999). However, after the FDA approved the use of fluoroquinolones in poultry production in 1995, fluoroquinolone-resistant *C. jejuni* were detected in both poultry and human isolates. The Minnesota Department of Health completed an analysis of *C. jejuni* isolates from humans and retail poultry products and found that the proportion of fluoroquinolone-resistant *C. jejuni* isolated from humans increased from 1.3% in 1992 to 10.2% in 1998 (following the 1995 fluoroquinolone approval) (Smith et al. 1999). In contrast, in Australia, where fluoroquinolones have never been approved for use in animal agriculture, no fluoroquinolone resistance has been detected in *C. jejuni* isolated from domestically acquired human infections (Unicomb et al. 2003).

### Variant Creutzfeldt-Jakob disease

Beyond bacterial infections, a chronic human health risk that has been linked to animal feeding practices is variant Creutzfeldt-Jakob disease (vCJD), a novel human neuro-degenerative prion disease that is currently untreatable and fatal (Collinge 1999). vCJD was first described in 1995 in two teenagers in the United Kingdom and was believed to be caused by infection with the causative agent of BSE or mad cow disease (Smith 2003). Molecular strain-typing studies and experimental transmission studies in mice published in 1996 and 1997 confirmed that vCJD is caused by the same prion strain that causes BSE (Collinge 1999).

The primary routes of human exposure to prions remain debatable; however, the most likely route is through the ingestion of beef derived from cattle that were infected when rendered animal proteins from diseased cattle were included in their feed. It is hypothesized that the UK population may have experienced the highest exposures to BSE from 1989 to 1990, when the incidence of BSE was still increasing in cattle and specific bans on high-risk rendered bovine products were still being implemented (Collinge 1999). From 1995 to 2002, there were 121 fatalities out of 129 diagnosed cases in the United Kingdom (Smith 2003). To date, domestically-acquired human cases of vCJD have not been identified in the United States. However, since BSE was first identified in the United States in 2003, the Centers for Disease Control and Prevention (CDC) have enhanced national surveillance for all types of CJD in the United States through the analysis of multiple cause-of-death data derived from death certificates (CDC 2005). Active CJD surveillance is also being implemented through the Emerging Infections Programs established in four sites across the United States (CDC 2005).

### Arsenic-related human health risks

Because inorganic As^III^ and As^V^ are known human carcinogens, there is considerable concern regarding human exposures to these compounds. Chronic arsenic exposures occurring through the ingestion of contaminated drinking water and dietary sources have resulted in skin cancers, lung cancers, bladder cancers, and prostate cancers, as well as hypertensive heart disease and nephritis [World Health Organization (WHO) 2001]. Although several research groups have begun to elucidate the effects of arsenic use in animal feed on environmental concentrations of arsenic in areas where animal waste has been land-applied (Bednar et al. 2003; Garbarino et al. 2003; Jackson et al. 2006; Stolz et al. 2007), only one study to date has explored how the presence of arsenic in U.S. meat products could potentially impact the health of consumers (Lasky et al. 2004).

Lasky et al. (2004) determined concentrations of total arsenic in poultry samples using data from the USDA Food Safety and Inspection Service, National Residue Program. National chicken consumption data were then used to quantify exposures to total arsenic, inorganic arsenic, and organic arsenic resulting from the consumption of poultry meat. The findings of this study indicated that individuals who consume average amounts of poultry (60 g/day) could ingest 1.38–5.24 μg/day of inorganic arsenic from the ingestion of poultry alone (Lasky et al. 2004), an amount that represents a high proportion of the tolerable daily intake of inorganic arsenic recommended by the Joint Food and Agriculture Organization (FAO)/WHO Expert Committee on Food Additives (2 μg/kg/day) (WHO 1983). Clearly, additional studies are necessary to further understand the associations between the ingestion of arsenic-contaminated meat and cancer risk.

### Mycotoxin-related human health risks

There are numerous peer-reviewed studies regarding human health effects associated with exposures to mycotoxins. These effects range from carcinogenic and nephrotoxic health effects to dermonecrotic and immunosuppressive health effects (Orriss 1997). Although the main route of human exposure to mycotoxins has been identified as the direct ingestion of contaminated cereals and grains (Orriss 1997), but there are few and conflicting studies about whether the ingestion of meat, milk, and eggs originating from mycotoxin-exposed food-production animals is a significant exposure pathway for mycotoxins among humans.

Researchers from the USDA Division of Epidemiology and Surveillance, have articulated that the ingestion of mycotoxin-contaminated animal-based food products could pose a concern to public health (Hollinger and Ekperigin 1999). Several studies have identified elevated levels of aflatoxin M1 and other mycotoxins in cow milk (Ghidini et al 2005; Sorensen and Elbaek 2005). In addition, in a study in which pigs were fed 100 mg/day fumonisin B1 for 5–11 days, mean fumonisin levels in edible muscle tissues were 43 μg/kg (Meyer et al. 2003). Although fumonisin levels administered to pigs in this study were significantly higher than levels ingested under normal agricultural conditions, the findings suggest that the consumption of meat from animals inadvertently exposed to elevated levels of fumonisin in feed could be a potential pathway for human exposure to these toxins. Others have found that trace levels of ochratoxin A in pork and poultry samples were likely to pose insignificant risks to consumers (Guillamont et al. 2005; Jorgensen 1998).

### PCDD-, PCDF-, and PCB-related human health risks

Many studies have indicated that animal-based food products (including fish and dairy products) are the largest dietary contributors to PCDD, PCDF, and PCB exposures in the U.S. population (Huwe and Larsen 2005; Schecter et al. 1994). Schecter et al. (1994) estimated daily dioxin TEQ intakes associated with the ingestion of dairy, meat, and fish by testing samples collected from a grocery store in upstate New York. When combined with 1986 U.S. food consumption rates, these estimates translated to an average daily dioxin TEQ intake ranging from 18 to 192 pg TEQ for an adult weighing 65 kg (Schecter et al. 1994). In another study that analyzed beef, pork, and poultry samples collected from nine cities across the United States, the estimated daily dietary intake ranged from 5.3 to 16.0 pg TEQ (Huwe and Larsen 2005). These levels represent a considerable portion of the tolerable daily intake for dioxin (TCDD; 1–4 pg/kg body weight) recommended by the WHO (1999).

Chronic exposures to PCDDs, PCDFs, and PCBs can result in adverse health effects ranging from cancers to impairments in the immune system, endocrine system, and reproductive organs (WHO 1999). Animal-based food products are known to be major dietary sources of human exposure to dioxin-like compounds; yet, the specific role that contaminated animal feeds play in this exposure pathway is unclear.

## Conclusions

Food-animal production in the United States has changed markedly in the past century, and these changes have paralleled major changes in animal feed formulations. While this industrialized system of food-animal production may result in increased production efficiencies, some of the changes in animal feeding practices may result in unintended adverse health consequences for consumers of animal-based food products.

Currently, the use of animal feed ingredients, including rendered animal products, animal waste, antibiotics, metals, and fats, could result in higher levels of bacteria, antibiotic-resistant bacteria, prions, arsenic, and dioxin-like compounds in animals and resulting animal-based food products intended for human consumption. Subsequent human health effects among consumers could include increases in bacterial infections (antibiotic-resistant and nonresistant) and increases in the risk of developing chronic (often fatal) diseases such as vCJD.

Nevertheless, in spite of the wide range of potential human health impacts that could result from animal feeding practices, there are little data collected at the federal or state level concerning the amounts of specific ingredients that are intentionally included in U.S. animal feed. In addition, almost no biological or chemical testing is conducted on complete U.S. animal feeds; insufficient testing is performed on retail meat products; and human health effects data are not appropriately linked to this information. These surveillance inadequacies make it difficult to conduct rigorous epidemiologic studies and risk assessments that could identify the extent to which specific human health risks are ultimately associated with animal feeding practices. For example, as noted above, there are insufficient data to determine whether other human food-borne bacterial illnesses besides those caused by *S. enterica* serotype Agona are associated with animal feeding practices. Likewise, there are insufficient data to determine the percentage of antibiotic-resistant human bacterial infections that are attributed to the non-therapeutic use of antibiotics in animal feed. Moreover, little research has been conducted to determine whether the use of organo-arsenicals in animal feed, which can lead to elevated levels of arsenic in meat products (Lasky et al. 2004), contributes to increases in cancer risk.

In order to address these research gaps, the following principal actions are necessary within the United States: *a*) implementation of a nationwide reporting system of the specific amounts and types of feed ingredients of concern to public health that are incorporated into animal feed, including antibiotics, arsenicals, rendered animal products, fats, and animal waste; *b*) funding and development of robust surveillance systems that monitor biological, chemical, and other etiologic agents throughout the animal-based food-production chain “from farm to fork” to human health outcomes; and *c*) increased communication and collaboration among feed professionals, food-animal producers, and veterinary and public health officials.

## Figures and Tables

**Table 1:** Animal feed ingredients that are legally used in U.S. animal feeds.^a^

Origin, raw material	Examples
Plant	
Forage	Alfalfa meal and hay, Bermuda coastal grass hay, corn plant, and soybean hay
Grains	Barley, corn (organic and genetically modified), oats, rice, sorghum, and wheat
Plant protein products	Canola meal, cottonseed cakes and meals, peanut meal, safflower meal, and soybean (organic and genetically modified) feed and meal
Processed grain by-products	Distillers products, brewers dried grains, corn gluten, sorghum germ cake and meal, peanut skins, and wheat bran
Fruit and fruit by-products	Dried citrus pulp, apple pomace, and pectin pulp
Molasses	Beet, citrus, starch, and cane molasses
Miscellaneous	Almond hulls and ground shells, buckwheat hulls, legumes and their by-products, and other crop by-products
Animal	
Rendered animal protein from the slaughter of food production animals and other animals	Meat meal, meat meal tankage, meat and bone meal, poultry meal, animal by-product meal, dried animal blood, blood meal, feather meal, egg-shell meal, hydrolyzed whole poultry, hydrolyzed hair, bone marrow, and animal digest from dead, dying, diseased, or disabled animals including deer and elk
Animal waste	Dried ruminant waste, dried swine waste, dried poultry litter, and undried processed animal waste products
Marine by-products	Fish meal, fish residue meal, crab meal, shrimp meal, fish oil, fish liver and glandular meal, and fish by-products
Dairy products	Dried cow milk, casein, whey products, and dried cheese
Mixed	
Fats and oils	Animal fat, vegetable fat or oil, and hydrolyzed fats
Restaurant food waste	Edible food waste from restaurants, bakeries, and cafeterias
Contaminated/adulterated food	Food adulterated with rodent, roach, or bird excreta that has been heat treated to destroy pathogenic organisms
Other	
Antibiotics	Tetracyclines, macrolides, fluoroquinolones, and streptogramins
By-products of drug manufacture	Spent mycelium and fermentation products
Arsenicals	Roxarsone and arsanilic acid
Other metal compounds	Copper compounds and metal amino acid complexes
Nonprotein nitrogen	Urea, ammonium chloride, and ammonium sulfate
Minerals	Bone charcoal, calcium carbonate, chalk rock, iron salts, magnesium salts, and oyster shell flour
Vitamins	Vitamins A, D, B_12_, E, niacin, and betaine
Direct-fed organisms	*Aspergillis niger, Bacillus subtilis, Bifidobacterium animalis, Enterococcus faecium,* and yeast
Flavors	Aloe vera gel concentrate, ginger, capsicum, and fennel
Enzymes	Phytase, cellulase, lactase, lipase, pepsin, and catalase
Additives generally regarded as safe (GRAS)	Acetic acid, sulfuric acid, aluminum salts, dextrans, glycerin, beeswax, sorbitol, and riboflavin
Preservatives	Butylated hydroxyanisole (BHA) and sodium bisulfite
Nutraceuticals	Herbal and botanical products
Plastics	Polyethylene roughage replacement

a Data adapted from AAFCO (2004).

**Table 2:** Biological, chemical, and other etiologic agents detected in animal feed and their potential human health impacts.

Etiologic agent	Examples	Potential human health impacts	References
Bacteria	*Salmonella* spp., *E. coli* O157:H7	Bacterial infections^a^	Angulo 2004; Crump et al. 2002; Davis et al. 2003
Antibiotic-resistant bacteria^b^	*E. faecium, E. coli, C. jejuni*	Antibiotic-resistant bacterial infections^a^	Aarestrup et al. 2000; Dargatz et al. 2005; Schwalbe et al. 1999; Sorensen et al. 2001
Prions	Causative agent of BSE	vCJD^c^	Gizzi et al. 2003; Smith 2003
Arsenicals	Roxarsone, As^III^, As^V^	Increased human exposures to inorganic arsenic that may contribute to increases in cancer risk^a^	Chapman and Johnson 2002; Lasky et al. 2004
Mycotoxins	Aflatoxins, ochratoxins, fumonisins, trichothecenes	Increased human exposures to mycotoxins that may contribute to increases in cancer and noncancer risks^a^	Bhat and Vasanthi 1999; Hussein and Brasel 2001
Dioxins and dioxin-like compounds	PCDDs, PCDFs, PCBs	Increased human exposures to dioxin-like compounds that may contribute to increases in cancer and noncancer risks^a^	Eljarrat et al. 2002; Fries 1995; Huwe and Larsen 2005

vCJD, variant Creutzfeldt-Jakob disease.

a Insufficient data are available to fully understand the magnitude of potential human health impacts associated with contaminated animal feed.

b Includes antibiotic-resistant bacteria initially present in animal feed due to contaminated feed ingredients, and antibiotic-resistant bacteria resulting from the nontherapeutic use of antibiotics in feed.

c Domestically acquired human cases of vCJD have not been documented in the United States.
